# Validation of Neuphony 8-channel EEG flex cap: a comparative study with BioSemi 64-channel EEG system

**DOI:** 10.3389/fnins.2025.1670860

**Published:** 2026-01-12

**Authors:** Ankita Rani, Amrendra Singh, Sudhakar Mishra

**Affiliations:** 1 National Neuroimaging Facility, Centre of Behavioral and Cognitive Sciences, University of Allahabad, Prayagraj, India; 2 Department of Artificial Intelligence, Sardar Vallabhbhai National Institute of Technology, Surat, India

**Keywords:** attention, EEG, ERP, signal to noise ratio, validation, wearable device

## Abstract

**Background:**

Electroencephalography (EEG) is a widely used tool in neurological assessment and cognitive research due to its reliability, high temporal resolution, and non-invasive nature. While portable EEG systems offer improved accessibility, many rely on electrode caps or extensive electrode arrays, which limit ease of use and portability in applied settings. The present study aimed to evaluate whether the 8-channel wearable Neuphony EEG flex cap can reliably capture key event-related potential (ERP) components such as P300 and mismatch negativity (MMN), while also providing robust measures of continuous EEG activity.

**Methods:**

This study involved 25 healthy participants, with an average age of 19.72 ± 0.8 years. EEG recordings were obtained using the Neuphony Flex Cap and BioSemi system while subjects engaged in the following tasks: resting state, auditory oddball, and visual discrimination tasks. Participants were instructed to keep their eyes open and concentrate on a cross during the resting state recording. Two distinct sound tones were played during the auditory oddball task to elicit P300 and MMN responses. For the visual discrimination task, participants were asked to respond upon seeing either an M or a W among distractors. Accuracy and reaction times were recorded for both tasks.

**Results:**

We evaluated the average relative PSD of the Neuphony EEG flex cap and BioSemi Active Two system across the entire frequency range (1–30 Hz), revealing a significant difference in the 8 to 13 Hz band. A repeated measures ANOVA showed a significant main effect of device for P300 amplitude [*F*(1, 24) = 271.59, *p* < 0.001, ηp^2^ = 0.919] and P300 latency [*F*(1, 24) = 8298.6, *p* < 0.001, partial η^2^ = 0.997] reflecting overall differences in recording between devices. Similarly for Late Positive Complex (LPC), a repeated measure ANOVA showed a significant main effect of device for amplitude [*F*(1, 24) = 16.44, *p* = 0.00046, partial η^2^ = 0.406] and latency [*F*(1, 24) = 7094.90, *p* < 0.001, partial η^2^ = 0.997].

**Discussion:**

This research indicated that it is feasible to obtain fair-quality EEG data for research purposes using the Neuphony EEG flex cap. However, the strength of the frequency band between 8 and 13 Hz showed variability, as did the event-related peaks. Event-related potentials (ERPs) were comparable for both devices, with a slight consistent delay in the 8-channel Neuphony EEG flex cap, potentially due to Bluetooth signal transmission.

## 1 Introduction

A highly efficient technique for identifying and diagnosing various brain disorders and abnormalities is electroencephalography, commonly known as EEG. It has consistently been regarded as the standard in neurological evaluation due to its reliability, quickness, and user-friendliness (Siuly et al., 2016). The utilization of EEG in cognitive neuroscience and various applied fields has seen a substantial surge, driven by its non-invasive nature, high temporal resolution, and relatively low cost compared to other neuroimaging techniques (Light et al., 2010). EEG stands out as a widely employed non-invasive tool for assessing the brain’s electrophysiological activity. It was initially employed to observe and record electrical signals generated by brain activity, thereby assisting in clinical assessments and diagnoses (St Louis et al., 2016). The technique’s evolution has been marked by advancements in signal processing, electrode technology, and analytical methods, enabling researchers to investigate a broad spectrum of cognitive processes, including attention, memory, language, and decision-making (Liu et al., 2023).

In the last 20 years, the use of EEG has expanded significantly, now including fields such as monitoring motor rehabilitation, assessing infant neurodevelopment, evaluating cognitive decline (such as in Alzheimer’s), sports training, analyzing mental states, measuring cognitive load, and developing brain-computer interfaces (Vasconcelos et al., 2021). These evolving applications necessitate specific EEG equipment characteristics to maximize their potential in real-world mobile settings, including portability, rapid setup, comfort, and unobtrusiveness. The convergence of technological advancements and methodological refinements has elevated EEG to a prominent position in understanding brain function in both healthy individuals and clinical populations (Kennett, 2012). Although most systems are equipped with software development kits that researchers can use to extract raw data for their investigations, there has been a lack of substantial efforts to validate the effectiveness of these platforms for ERP research (Badcock et al., 2013; Badcock et al., 2015; Debener et al., 2012; Maskeliunas et al., 2016). Furthermore, much of the previous portable EEG research has depended on electrode caps or extensive electrode arrays, which complicate participant setup and, as a result, reduce the system’s portability and effectiveness. This limitation restricts the systems’ usability in specific contexts.

From a technical perspective, several factors contribute to the challenges of using the ERP technique in affordable, non-standard, research-grade equipment. The main issue is data quality, particularly whether budget-friendly systems can deliver sampling rates (≥ 250 Hz) and data quality (i.e., free from noise and artifacts) that are adequate for standard ERP analysis. Since ERP components are typically linked to the examination of particular electrode locations, the matter of experimental time also raises two additional significant concerns: first, how to “mark” the data for subsequent ERP analysis, and second, the challenge of non-standard electrode placements for evaluation (Krigolson et al., 2017). The number of channels (Srinivasan et al., 1998) on an EEG device is just one of numerous factors that influence its precision. Other crucial variables encompass the method (invasive vs. noninvasive) used to establish contact between the electrode and the scalp, the type of electrode (dry vs. gel-based) (Coles et al., 1986), the quality of the contact (“impedance” range kΩ vs. MΩ) (Kutas, 1997), the configuration of the amplifier (Krigolson et al., 2017) (passive vs. active), the placement of the electrodes, including the reference electrodes, and various other subtle influencing factors such as scalp perspiration and hair. Selecting the electrode positions, along with the references, is also crucial. An affordable, commercially available, wearable EEG headset featuring eight electrodes two prefrontal (Fp1, Fp2), three frontal (F3, Fz, F4), one parietal (Pz), and two occipital (O1, O2) dry active EEG channels is the Neuphony flex cap (Pankhtech India Pvt. Ltd.). To our knowledge, it has yet to be validated for ERP research (i.e., time domain) as well as for power spectra (frequency domain) analysis.

The primary objective of the present study is to compare the P300 amplitude and latency obtained using a newly developed dry electrode EEG system with those recorded by a conventional wet electrode device. This comparison aims to evaluate whether the two systems yield comparable ERPs, thereby establishing the reliability of the dry electrode system in capturing cognitive neural responses. Among the most widely studied ERPs are the P300 and mismatch negativity (MMN), both of which are sensitive to stimulus frequency and are typically elicited using the oddball paradigm (Squires et al., 1975). Accordingly, this paradigm was employed in the present study to validate the performance of the dry electrode EEG device. A secondary aim of the study is to assess the signal-to-noise ratio (SNR) between the two devices, based on the quality of ERP waveforms. In addition, we compare the spectral power across canonical frequency bands (delta, theta, alpha, beta, and gamma) to further establish the robustness of the dry electrode system in capturing ongoing neural oscillations alongside ERPs. We also investigated whether the dry electrode system can reliably capture the late positive complex (LPC), a key component reflecting cognitive evaluation processes, with accuracy similar to that of the conventional EEG. Taken together, the overarching goal is to validate the performance of the dry electrode EEG device and examine its potential to serve as a practical and reliable alternative to conventional wet electrode systems. Moreover, to provoke a broad spectrum of late ERP components, we examined LPC induced by discriminative stimuli, as previously demonstrated (Friedman et al., 1978). All three components were chosen because they each assess a range of cognitive and perceptual events and are generally quite significant (in terms of μV effect size).

## 2 Materials and methods

The study was approved by the Institutional Ethics Review Board (IERB), University of Allahabad (*Ref No. IERB/26/2025 dated 17/01/2025*) ensuring compliance with ethical research standards for studies involving human participants.

### 2.1 Participants

A total cohort consisting of 20-five healthy adults (12 females and 13 males; mean age: 19.72 ± 0.8 years) was enlisted for the investigation. The size of the participant group was ascertained through *a priori* determination of sample size given *α* = 0.025 (based on Bonferroni corrected value of alpha given two outcomes were designated as primary), power = 0.80, and effect size = 0.78 using G power version 3.1.9.7. The effect size was calculated based on partial η^2^ = 0.38 reported in previous study conducted by Ehrhardt et al. (2024) under Bonferroni corrected testing. Prior to their involvement, written informed consent was procured. Each participant possessed either normal vision or vision corrected to normal and exhibited no documented history of neurological or psychiatric disorders. Participants were required to complete a self-administered questionnaire assessing their levels of depression (Beck Depression Inventory), anxiety [State–Trait Anxiety Inventory (STAI)], and impulsivity (full UPPS-P scale). All participants engaged in the identical task across both devices in two sessions, with a minimum interval of 1 week to mitigate any potential practice effects. The sequence of data collection was systematically counterbalanced across the cohort, with the initial 50% of participants executing the task first on the Biosemi device and subsequently on the Neuphony device.

### 2.2 Experimental design

The study was performed in a soundproofed setting. Electroencephalographic (EEG) data were collected using the Neuphony Flex Cap along with the BioSemi system. The Neuphony cap successfully recorded electrical activity from eight electrodes (Fp1, Fp2, F3, Fz, F4, Pz, O1, O2), while the BioSemi system gathered data from all 64 channels. Three experimental tasks were designed to assess the signal quality, specifically the resting state task, the auditory oddball task, and the visual discrimination task. EEG recordings were taken during each of the three tasks. The same group of participants performed the tasks using both the Neuphony EEG Flex Cap system and the BioSemi Active Two system, with a one-week gap between sessions. Simultaneous recording was not possible due to the identical electrode placement and configuration of the EEG caps.

#### 2.2.1 Resting state

Participants were positioned in a soundproof, dimly lit chamber in front of a 19-inch LCD monitor located at a distance of 25 cm. They were instructed to maintain an open gaze while concentrating on a fixation cross displayed at the center of the screen and to refrain from engaging in any extraneous bodily movements. The recording of the resting state was conducted for a minimum duration of 5 min for each system (Ratti et al., 2017).

#### 2.2.2 Auditory oddball task

**Figure 1:**
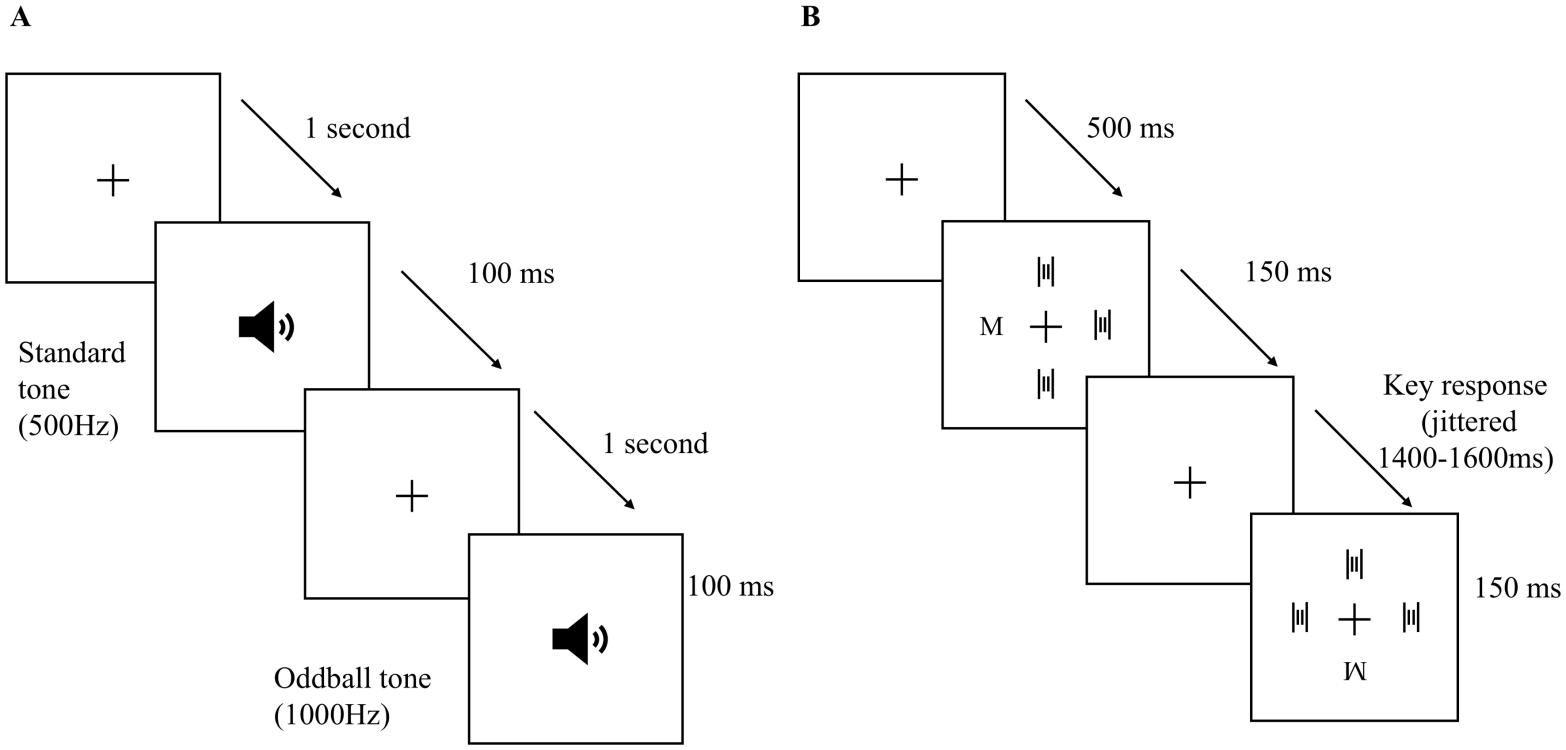
Experimental paradigms for auditory and visual tasks. **(A)** Auditory oddball task: Participants passively listened to a sequence of tones consisting of frequent *standard tones* (500 Hz) and infrequent *oddball tones* (1,000 Hz), each lasting 100 ms, with a 1 s interstimulus interval. **(B)** Visual discrimination task: Each trial began with a fixation cross (500 ms) followed by a 150 ms display containing one target letter (*M* or *W*) and three distractors. Participants were instructed to identify the target letter via a keypress during a response window jittered between 1,400–1,600 ms.

The experimental tasks were carried out by participants using a standard USB keyboard while placed in an acoustically treated setting, situated in front of a 19-inch LCD monitor. The stimuli were presented through the Psychophysics toolbox extension (MATLAB 2019b). Participants were directed to focus on the black fixation cross (MATLAB RGB value = [0 0 0]) positioned at the center of the display, while two different auditory stimuli the standard tone (500 Hz) and the oddball tone (1,000 Hz) were played through two speakers (85 dB SPL intensity) set about 1 meter apart, facing the participants (Furdea et al., 2009). A total of 300 trials were conducted, each consisting of a reaction time period of 1 s and a sound tone length of 100 ms (Figure 1A). Thirty percent of the auditory stimuli were identified as oddball tones, while 70% were identified as standard tones, a ratio considered sufficient to provoke a P300 response. Participants were instructed to press the space key solely for oddball tones and to avoid any unnecessary body movements or excessive blinking during the task’s performance. The reaction times and accuracy of the correct responses (oddball) were carefully recorded.

#### 2.2.3 Visual discrimination task

Participants performed the experimental tasks utilizing a standard USB keyboard while seated in an acoustically insulated darkened environment, situated before a 19-inch LCD computer monitor at a distance of 110 cm, with the response keys positioned beneath their left and right index fingers. The participants’ visual focus was directed such that the fixation point was aligned with the midpoint of their horizontal visual field. In this experimental paradigm, a total of 384 trials were conducted across six distinct blocks (64 trials per block). During each trial, an array of four stimuli was presented for 150 ms, arranged above and below, as well as to the left and right of the fixation cross. The horizontal or vertical spacing between each stimulus and the fixation cross was approximately 3.6 degrees. In every trial, a letter stimulus (either M or W) was displayed alongside three distractors (two long and two short vertical bars), as demonstrated in Figure 1B. Participants were directed to respond by pressing a key (the left Ctrl key for the letter M and the right Ctrl key for the letter W) immediately upon the appearance of the target letter, while maintaining their focus on the central fixation cross on the screen. The letter stimuli functioned as target stimuli with a 50% likelihood of manifesting (either M or W) at any of the four designated positions (top, bottom, left, and right). The emergence of the target stimuli at these four locations was randomized. To mitigate the potential influence of stimulus anticipation, the stimulus-onset asynchrony was randomly varied between 1,400 and 1,600 ms, during which participants were required to respond to the target letters. To facilitate participants’ acclimatization to the task requirements, several training trials were administered at the commencement of the experiment. A temporal representation of the experimental procedure is illustrated in Figure 1B.

### 2.3 EEG data acquisition

#### 2.3.1 Biosemi active two measurement system

EEG data were recorded using Active View Software (Version 3.2, BioSemi) in conjunction with 64 active Ag/AgCl wet gel electrodes, which were affixed to a custom-fitted cap adhering to the standard 10–20 electrode placement paradigm, alongside two passive electrodes designated as common mode sense (CMS) and driven right leg (DRL). The DRL and CMS electrodes function as integral components of a feedback mechanism. The closure of this feedback loop is requisite to facilitate the stabilization of the subject’s electrical potential in proximity to the reference of the AD-box. The offsets associated with each active electrode were maintained within the range of ±40 mV during quiescent states. Electrode offset serves as an indicator of the half-cell potential at the interface between the electrode gel and the scalp. In Biosemi, electrode offset is used instead of impedance as a measure of electrode to scalp contact quality. To ensure compliance with the offset criterion, participants were instructed to maintain a clean and oil-free scalp prior to the commencement of the study. The electrodes affixed to the cap were initially referenced to mastoid electrodes, which were not included in the subsequent analysis. The data acquisition was initially conducted at a sampling rate of 2048 Hz, and the signals were subjected to amplification and filtering via a fixed first-order analog anti-aliasing filter with a −3 dB cut-off frequency at 3.6 kHz.

#### 2.3.2 Neuphony EEG flex cap

During the evaluation of signal quality, electroencephalogram (EEG) signals were acquired using a Neuphony EEG flex cap’s application interface, employing Lab Recorder software in conjunction with eight Ag/AgCl coated electrodes that were firmly affixed to the flex cap. The electrodes were strategically positioned in accordance with the international 10–20 system at locations Fp1, Fp2, F3, Fz, F4, Pz, O1, and O2, with A1 and A2 designated as the reference electrodes. Following each pertinent stimulus, we transmitted the experimental data alongside the Bluetooth EEG data (version 4.2) to the lab recorder utilizing the Lab Streaming Layer (MATLAB 2019b). The recorded EEG data was sampled at a frequency of 250 Hz. The device employs a proprietary impedance-check algorithm that operates within an internally defined acceptable range. The exact impedance threshold was not directly quantified before recording. Instead, the system provided a qualitative indicator (green signal) to confirm when electrode skin contact met its internal impedance criteria, after which recording is permitted. Consequently, we were unable to report precise impedance values prior to data acquisition, and impedance verification was restricted to the device’s built-in acceptance check. In order to quantify the impedance, we did a *post hoc* estimation of impedance which gave an impedance of median value of 124.60 kΩ (88.40–177.58 kΩ). It was estimated using the Johnson–Nyquist thermal noise relation (Johnson, 1928; Nyquist, 1928), which links the mean-square voltage noise across a resistor to its resistance, bandwidth, and temperature. This approach has been previously applied in biomedical instrumentation to infer electrode skin interface impedance (Webster, 2014; Huigen et al., 2002).

### 2.4 EEG data preprocessing

The MATLAB scripts utilizing the EEGLAB (version 2024.2) toolbox (Delorme and Makeig, 2004) were run on MATLAB R2022a (MathWorks Inc., Natick, United States) for all analyses performed. Most of the preprocessing steps (Chaddad et al., 2023) were similar for the EEG data collected from both systems, except for the downsampling process, which was only applied to the datasets from the BioSemi Active Two measurement system in order to normalize the sampling rate across the devices. The collected data were then imported into EEGLAB, where channel locations were defined, and subsequently, the data were subjected to band-pass filtering within the frequency range of 1 Hz to 30 Hz using a finite impulse response (FIR) filter, followed by resampling at 250 Hz (only for BioSemi data) with a polyphase anti-aliasing filter, as specified in the EEGLAB functions pop_eegfilt and pop_resample, respectively. The dataset was meticulously examined for segments containing artifacts, which were subsequently eliminated using the EEG function clean_rawdata. Artifact Subspace Reconstruction (ASR) correction for bad bursts was applied with a maximum allowable standard deviation of 20 within a 0.5-s window. This method estimates the statistical distribution of clean EEG from a calibration period and marks a data window w for removal of its variance exceeds a threshold relative to calibration variance,

$$\frac{\mathrm{Var}(w)}{\mathrm{Va}r_{\text{calib}}}>\theta ,\text{with}\;\theta =20$$

This means that any data segments showing a deviation greater than this limit compared to the calibration data were marked as missing and thus discarded. Issues such as flat channels, weakly correlated channels, and line noise interferences were not detected by the previously mentioned voltage-based criteria, although they were successful in identifying voltage drifts. To ensure high-quality EEG signals, artifact rejection was performed at both the channel and data-segment levels. At the channel level, three statistical criteria were applied. First, channels were considered unreliable if they exhibited flat-line activity for extended periods. Formally, a channel $x_{c}(t)$ was marked as bad if its signal remained constant for more than 6 s, such that

$$\max_{t\in [t0,t0+6]}\;x_{c}\;(t)-\min_{t\in [t0,t0+6]}\;x_{c}\;(t)<\varepsilon ,$$

with ∈≈0μV , indicating a loss of effective recording. Second, channels were evaluated based on their correlation with the rest of the scalp field. A predicted signal ${\hat{x}}_{c}(t)$ was estimated from the other channels using robust regression, and the Pearson correlation coefficient was computed as

$$r=\frac{\mathrm{cov}(x_{c},{\hat{x}}_{c})}{\sigma_{x_{c}}\sigma_{{\hat{x}}_{c}}}$$

If this value fell below 0.8, the channel was excluded, as low correlation suggested poor spatial consistency with the surrounding electrodes. Finally, channels were examined for excessive contamination by line noise. For each channel, the power spectral density P(f) was estimated, and the relative strength of the line frequency ( $f_{\text{line}}=50\;\mathrm{Hz}$ ) compared to its neighboring frequency bands was calculated

$$R=\frac{P(f_{\text{line}})}{P(f_{\text{line}}\pm \mathrm{\Delta f})}$$

Channels were discarded when this ratio exceeded a value of 4, indicating disproportionate line interference. This steps were performed using the EEGLAB function clean_rawdata with criterions, flat line criterion: 6; channel correlation criterion: 0.8; line noise criterion: 4.

The data were then re-referenced to the average of all channels. RunICA was employed to decompose the CleanLine data through Independent Component Analysis (ICA) (Bell and Sejnowski, 1995; Amari, 1998; Lee et al., 1999), and ICLabel was used to classify the independent component processes that emerged (Pion-Tonachini et al., 2019). All independent components (ICs) flagged by ICLabel that indicated artifacts from the heart, muscles, eyes, or line noise, with a probability range of 0.9–1, were excluded. The continuous EEG data for each channel were then reconstructed by summing the scalp projections of the remaining ICs, which were expected to represent either cortical brain activity or other unclassified activities. To extract event-related potentials (ERPs) from the continuous dataset, we used the ERPLAB (version 12.0) toolbox. Each participant’s responses were compiled into a distinct event list, which included the corresponding trigger numbers. After assigning events to the correct bins using BINLISTER, we were able to extract epochs according to bin criteria. The epoching procedure was carried out in such a way that each participant’s corresponding ERPs were sorted into separate bins, with a pre-stimulus duration of 200 ms and a post-stimulus duration of 800 ms. The pre-stimulus baseline correction (−200 to 0 ms) was performed while averaging the ERP for each participant across the trials at each electrode. The methods used for epoching and ERP extraction were consistent across both devices.

### 2.5 Statistical analysis

All analyses were conducted using MATLAB (version 2022a). Statistical analyses were executed utilizing repeated-measures, within-subject Analysis of Variance (ANOVA), treating participants as a random variable and the levels of within-subject components as repeated measures. The primary outcomes of the study were P300 amplitude and P300 latency, both measured for standard and oddball stimuli across the two devices. Because two primary outcomes were evaluated, a Bonferroni correction was applied to control the familywise Type I error rate. Accordingly, all inferential tests pertaining to primary outcomes were evaluated against a corrected significance threshold of *α* = 0.025. When significant effects were observed, post-hoc paired-sample *t*-tests were conducted to compare Standard vs. Oddball stimuli within each device, and Biosemi vs. Neuphony within each condition. Because four post-hoc comparisons were performed for each primary outcome, a Bonferroni-adjusted threshold of α = 0.00625 (0.025/4) was applied to the post-hoc analyses. The Welch’s averaged modified periodogram method, available through the MATLAB function *pwelch* (window length, 2 s; overlap, 1 s), was employed to calculate the log PSD for the continuous data (Resting state) independently for each channel of the 10–20 System. To facilitate the comparison of PSDs across systems, frequency spectra were limited to under 30 Hz, and Pearson’s correlation coefficient was computed for log PSD across the 10–20 system.

### 2.6 Signal-to-noise ratio calculation

The signal-to-noise ratio (SNR) was calculated and compared for the ERP waveforms obtained in response to the auditory oddball paradigm across eight electrodes. To compare the quality of averaged ERP signals recorded from two EEG systems, Neuphony and Biosemi, across eight electrodes (Fp1, Fp2, F3, Fz, F4, Pz, O1, O2), the SNR was calculated. Each channel’s SNR was determined using the following formula:

$$\mathrm{SN}R_{\mathrm{dB}}=20\cdot \log_{10}(\frac{|\mu_{\text{signal}}|}{\sigma_{\text{noise}}})$$

where μ_signal_​ represents the mean amplitude of the ERP waveform within the signal window (250–500 ms), and σ_noise_ is the standard deviation of the baseline period (−200 to 0 ms), both defined relative to stimulus onset. This approach quantifies how strongly the evoked signal stands out from the background noise. The ERP data were loaded using *pop_loaderp*, and the SNR for each electrode was computed using a custom function, *compute_erp_snr*.

## 3 Results

### 3.1 Signal-to-noise ratio

A dependent samples *t*-test was conducted to compare SNR values between Neuphony and Biosemi devices across eight electrodes (Table 1). The results indicated that SNR obtained by Neuphony (7.81 dB ± 10.2 dB) did not differ significantly from Biosemi (5.24 dB ± 2.72 dB) [*t*(7) = 0.712, *p* = 0.499, CI (−5.96, 11.1)]. Although, Neuphony demonstrated good SNR values at central and posterior sites, including F3 (19.21 dB), Pz (14.66 dB), and O1 (19.75 dB) which is indication of good signal quality in these areas, the channel-to-channel variability is much higher than Biosemi. It also demonstrated negative SNR values at frontal sites such as Fp1 (−5.42 dB) and Fp2 (−4.83 dB), indicating noise contamination in those channels. Biosemi, on the other hand, consistently achieved positive SNR values across all electrodes, albeit at a lesser magnitude than Neuphony’s top channels. SNR values at Pz (8.08 dB) and O1 (9.17 dB) were relatively lower, while frontal electrode like Fp1 (2.93 dB) and Fp2 (5.82 dB) exhibited adequate SNR.

**Table 1:** Signal-to-noise ratio (SNR, dB) comparison between Neuphony and Biosemi EEG systems across eight electrodes.

Channel	Neuphony_SNR_dB	Biosemi_SNR_dB
Fp1	−5.41909	2.929218836
Fp2	−4.83387	5.81803069
F3	19.20956	0.966872851
Fz	7.427549	5.734946132
F4	11.77089	3.348424475
Pz	14.66208	8.077068557
O1	19.74579	9.166255427
O2	−0.09431	5.882419086

### 3.2 Resting state

**Figure 2:**
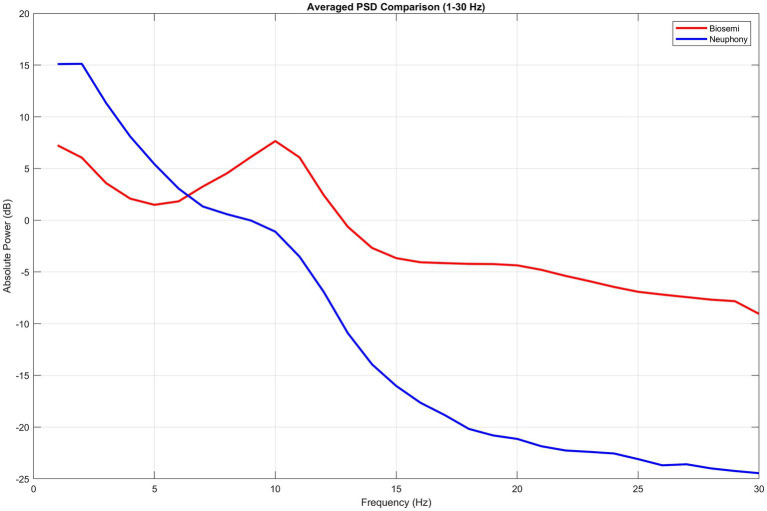
Averaged power spectral density (PSD) comparison between Biosemi and Neuphony EEG systems (1–30 Hz). The plot illustrates the absolute power (in dB) across frequency bands for the Biosemi (red) and Neuphony (blue) systems.

The absolute PSD curves of the preprocessed EEG obtained by both devices are shown in Figure 2. We assessed the average PSD of the Neuphony EEG flex cap and Biosemi Active Two system across the whole frequency band (1–30 Hz). All electrodes were considered for power calculation in case of Neurphony while only corresponding eight electrodes were taken into account for Biosemi device. We performed multiple dependent *t*-tests for each band to compare the difference in power of a particular band between the devices which indicated significant difference between power for each frequency band delta (1–4 Hz) [*t*(7) = −8.79, *p* < 0.001, 95% CI = −10.440, −6.015], theta (4–7 Hz) [*t*(7) = −4.06, *p* = 0.0048, 95% CI = −3.661, −0.966], alpha (8–13 Hz) [*t*(7) = 7.92, *p* = 0.0001, 95% CI = 5.306, 9.825], and beta (13–30 Hz) [*t*(7) = 20.32, *p* < 0.001], 95% CI = [13.491, 17.045]. Statistical metrics like *t*-values or signal-to-noise ratios can show that signal quality is not constant across frequencies but rather shows a frequency-dependent trend, frequently declining at lower frequencies and increasing at higher ones.

### 3.3 Auditory Oddball paradigm

#### 3.3.1 P300 component

**Figure 3:**
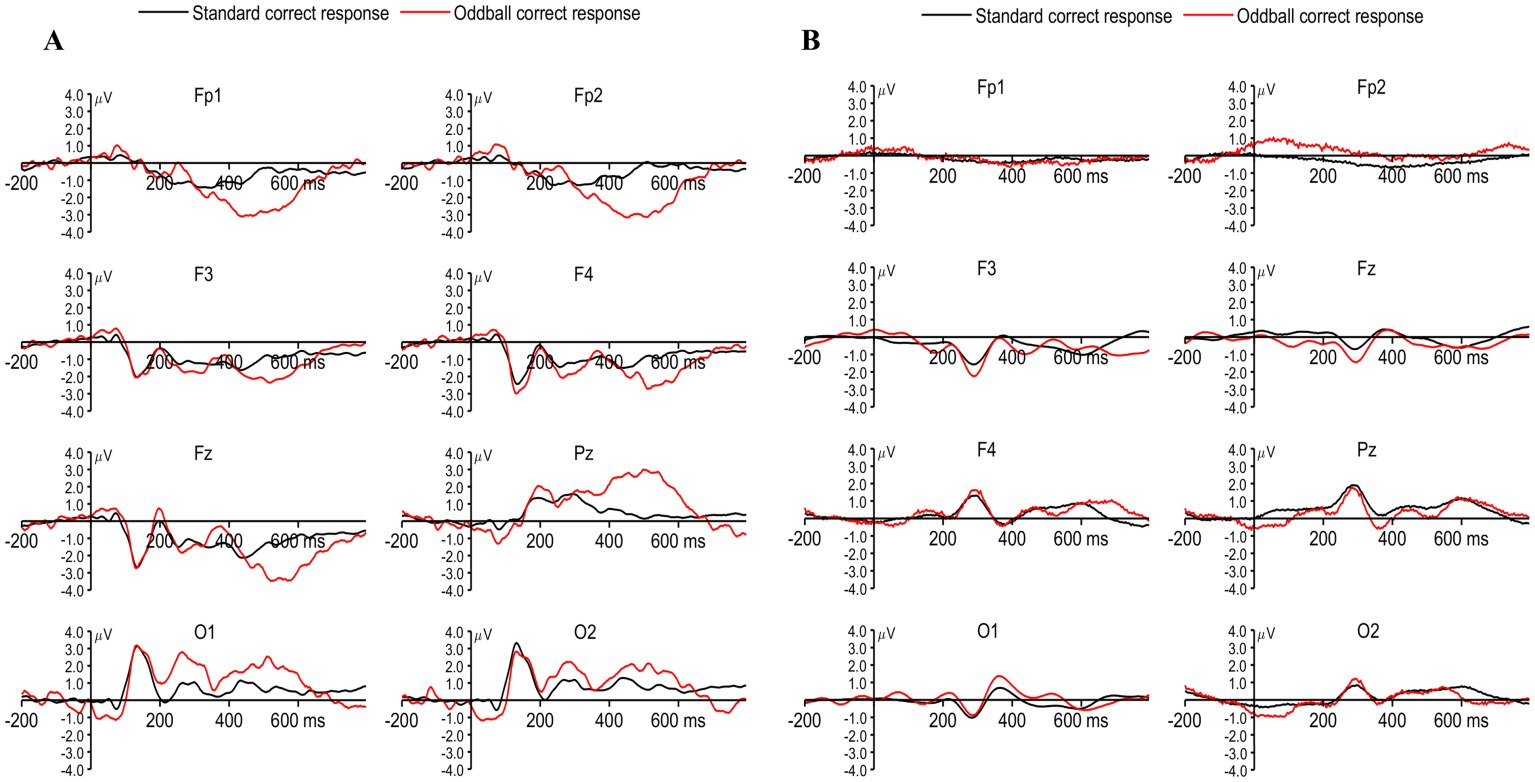
Grand-averaged event-related potentials (ERPs) for standard and oddball correct responses across eight electrodes for Biosemi and Neuphony EEG systems. **(A)** Biosemi ActiveTwo system. **(B)** Neuphony EEG Flex Cap. ERP waveforms represent standard (black) and oddball (red) tone responses at electrodes Fp1, Fp2, F3, F4, Fz, Pz, O1, and O2. Compared to standard trials, oddball stimuli elicited larger positive deflections, particularly over fronto-central sites, consistent with the characteristic P300 component.

**Figure 4:**
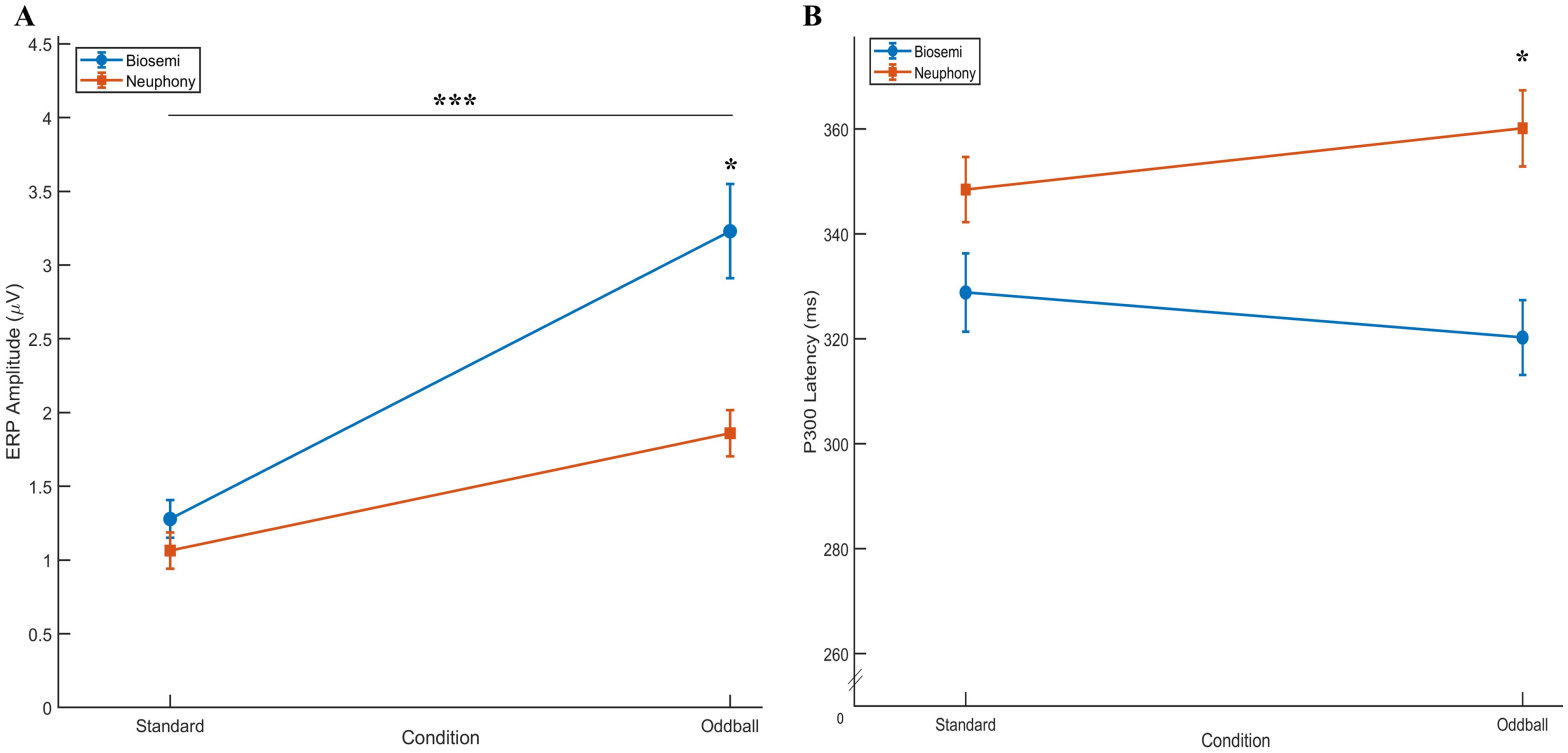
Comparison of P300 amplitude and latency across devices and conditions. **(A)** Mean P300 amplitude (μV) as a function of Condition (Standard vs. Oddball) and Device (Biosemi vs. Neuphony). Both devices show larger amplitudes for Oddball compared to Standard trials, consistent with the expected P300 effect, though Biosemi exhibited higher overall amplitudes. **(B)** Mean P300 latency (ms) for the same conditions and devices. While Biosemi demonstrated slightly shorter latencies overall, the latency patterns across conditions remained comparable between devices, indicating similar temporal sensitivity in detecting cognitive responses. Error bars represent ±1 SEM. Significance marker: “*” represents *p* < 0.05; “***” represents *p* < 0.001.

The averaged ERP curves with low frequency tone 500 Hz (standard) and high frequency tone 1,000 Hz (deviation) stimulations in the auditory oddball task, which was computed on EEG for Biosemi and Neuphony devices, are shown in Figures 3A,B, respectively. A 2 × 2 repeated measures ANOVA with factors Condition (Standard vs. Oddball) and Device (Biosemi vs. Neuphony) revealed a significant main effect of Condition, *F*(1, 24) = 252.14, *p* < 0.001, ηp^2^ = 0.913, indicating that ERP amplitudes were larger for Oddball than Standard trials. The main effect of Device was also significant, *F*(1, 24) = 271.59, *p* < 0.001, ηp^2^ = 0.919. Also, there was a significant Condition × Device interaction, *F*(1, 24) = 280.19, *p* < 0.001, ηp^2^ = 0.921 (Figure 4A). Because amplitude was one of the two primary outcomes, ANOVA effects were evaluated against the Bonferroni-corrected threshold (*α* = 0.025). All three ANOVA effects remained significant after correction. Post-hoc paired *t*-tests showed that the Condition effect was significant in both Biosemi [*t*(24) = −7.59, *p* < 0.001] and Neuphony [*t*(24) = −8.69, *p* < 0.001]. When comparing devices within each condition, there was no significant difference for Standard trials [*t*(24) = 1.15, *p* = 0.26], but Oddball trials were significantly larger in Biosemi than Neuphony [*t*(24) = 3.51, *p* = 0.002], which remained significant under the corrected threshold. These results indicate that the difference between Standard and Oddball ERP amplitudes depends on the device used, with very large effect sizes for all main effects and the interaction.

Similarly, 2 (Condition: Standard, Oddball) × 2 (Device: Biosemi, Neuphony) repeated-measures ANOVA was conducted on P300 latency across 25 subjects. The analysis revealed significant main effects of Condition, *F*(1, 24) = 5648.7, *p* < 0.001, partial η^2^ = 0.996, and Device, *F*(1, 24) = 8298.6, *p* < 0.001, partial η^2^ = 0.997. There was also a significant Condition × Device interaction, *F*(1, 24) = 6410.7, *p* < 0.001, partial η^2^ = 0.996, indicating that the interaction reflects device related differences in latency measurement rather than true condition related neural differences (Figure 4B). Latency was also a primary outcome, so ANOVA effects were evaluated against the corrected threshold (*α* = 0.025). All effects remained significant after correction. Post-hoc comparisons showed that latency did not differ significantly between Standard and Oddball within Biosemi [*t*(24) = 0.63, *p* = 0.535] or Neuphony [*t*(24) = −1.42, *p* = 0.168]. Latency did not differ between devices for Standard stimuli [*t*(24) = −1.62, *p* = 0.119], but was significantly different for Oddball stimuli [*t*(24) = −3.58, *p* = 0.002], which primarily drove the significant interaction.

#### 3.3.2 Mismatched negativity component

The MMN is the strongest in the temporal and frontal regions of topographic scalp maps, is triggered by abrupt changes in stimulation, and peaks 100–250 ms after the change onset (Sams et al., 1985). The MMN component waveform was calculated using a custom script on MATLAB 2022a. MMN represents the difference in ERP components between the two stimuli (oddball tones minus standard tones) (Williams et al., 2020; Gao et al., 2022). The passive MMN waveform’s significant negative deflection served as a representation of the MMN. When the MMN was present, it was calculated as the lowest voltage (a negative peak) in the interval throughout which the MMN waveform dropped below 0. MMN was obtained for both devices at each electrode location separately. The amplitude and latency of the MMN obtained for Biosemi and Neuphony are given in Tables 2, 3, respectively.

**Table 2:** MMN was calculated as the difference between oddball and standard tone responses at eight electrodes corresponding to the Neuphony montage.

Mismatch negativity (MMN) across electrodes for the Biosemi active two EEG system			
Electrodes	MMN_amplitude	MMN_Latency	*p*-value
Fp1	−1.2365	400	0.35896
Fp2	−0.63212	276	0.00019829
F3	−0.96351	100	1.79E-17
Fz	−0.79344	100	0.00016359
F4	−1.6946	400	0.1407
Pz	−0.62793	276	7.47E-05
O1	−0.7111	276	1.24E-10
O2	−1.2822	100	5.16E-07

Strong MMN responses were observed over fronto-central and occipital sites (F3, Fz, O1, O2), consistent with the typical scalp distribution, confirming reliable auditory deviance detection in the Biosemi system.

**Table 3:** MMN was computed as the difference waveform between oddball and standard tone responses across eight electrodes of the Neuphony EEG Flex Cap.

Mismatch Negativity (MMN) amplitudes and latencies across electrodes for the Neuphony EEG Flex Cap.			
Electrodes	MMN_amplitude	MMN_Latency	p value
Fp1	−0.26979	396	0.0045203
Fp2	0.34928	396	2.09E-55
F3	−0.67983	288	4.97E-11
Fz	−0.8086	116	7.35E-31
F4	−0.27184	240	1.90E-05
Pz	−0.81952	356	1.23E-23
O1	−0.079328	136	8.49E-19
O2	−0.55149	100	0.69135

The table presents mean MMN amplitudes (μV), corresponding peak latencies (ms), and statistical significance values. Prominent MMN responses with significant amplitudes were observed over fronto-central (F3, Fz, F4) and parietal (Pz) regions, consistent with the typical scalp distribution of MMN, indicating reliable auditory deviance detection in the Neuphony system.

### 3.4 Visual discrimination task

#### 3.4.1 LPC complex

**Figure 5:**
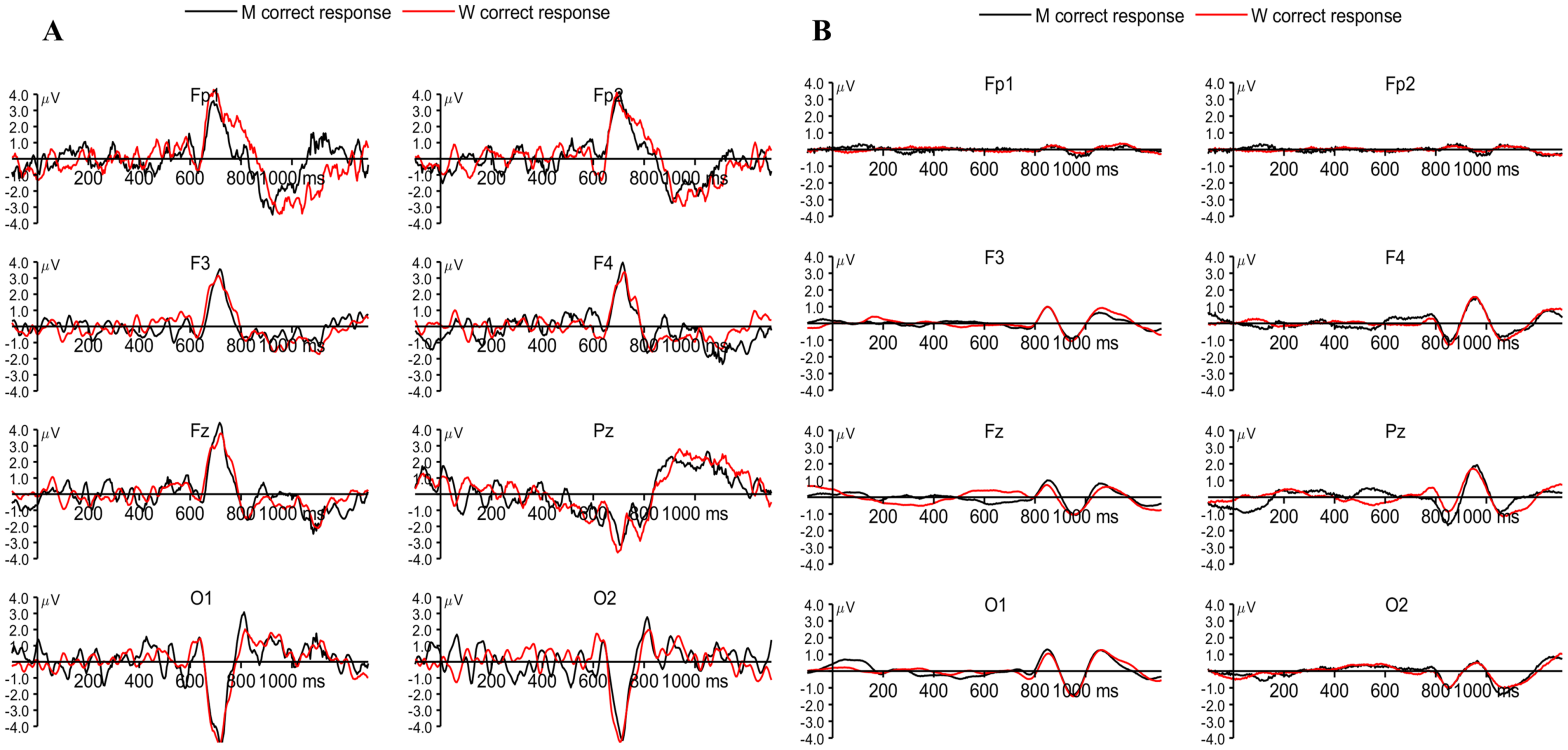
**(A)** Grand-averaged event-related potentials (ERPs) recorded using the Biosemi system for correct responses to target letters M (black) and W (red) across frontal (Fp1, Fp2, F3, F4, Fz), parietal (Pz), and occipital (O1, O2) electrodes. Prominent LPC components can be observed around 600–900 ms post-stimulus, particularly over frontal and parietal sites. **(B)** Corresponding ERPs recorded using the Neuphony system for the same conditions and electrode locations, showing similar but attenuated LPC responses.

We examined the late peaks obtained for both correct responses as well as incorrect responses, while no response trials were not considered for the ERP analysis across the device. Late components are the deflections obtained beyond the P300 component (Friedman et al., 1978). The LPC occurs approximately between 300 and 800 ms (Friedman et al., 1978; Squires et al., 1977). The LPC obtained by Biosemi and Neuphony are shown in Figures 5A,B, respectively. The comparison was made for LPC in a similar way to that of P300 between the devices. A 2 × 2 repeated-measures ANOVA was conducted with Condition (Correct vs. Incorrect responses) and Device (Biosemi vs. Neuphony) as within-subject factors on the LPC amplitude. The analysis revealed a significant main effect of Condition, *F*(1, 24) = 19.17, *p* = 0.0002, partial η^2^ = 0.444, indicating that LPC amplitudes were significantly higher for the correct condition compared to the incorrect condition. There was also a significant main effect of Device, *F*(1, 24) = 16.44, *p* = 0.00046, partial η^2^ = 0.406, suggesting that overall LPC amplitudes differed between the Biosemi and Neuphony systems. Importantly, the Condition × Device interaction was significant, *F*(1, 24) = 19.17, *p* = 0.0002, partial η^2^ = 0.444, indicating that the difference in LPC amplitude between correct and incorrect trials varied across the two devices. Post-hoc paired *t*-tests showed that the Condition effect was non-significant in both Biosemi [*t*(24) = −1.07, *p* = 0.297] and Neuphony [*t*(24) = 0.26, *p* = 0.796]. However, a significant device effect emerged, LPC amplitude was greater for Biosemi compared to Neuphony in both conditions. Specifically, in the correct condition, Biosemi recorded significantly higher LPC amplitude [*t*(24) = 2.23, *p* = 0.035], and this difference was more pronounced in the incorrect condition [*t*(24) = 4.12, *p* < 0.001]. These findings suggest that while task condition did not modulate LPC amplitude within devices, the recording device significantly influenced amplitude, with Biosemi consistently showing stronger LPC responses than Neuphony.

Similarly, 2 (Conditions) × 2 (Device: Biosemi vs. Neuphony) repeated-measures ANOVA was conducted on LPC latency across 25 subjects and the analysis revealed a significant main effect of condition *F*(1, 24) = 4,446.20, *p* < 0.001, partial η^2^ = 0.994, indicating that LPC latency significantly differed between the two task conditions (correct vs. incorrect responses). A significant main effect of Device was also observed, *F*(1, 24) = 7,094.90, *p* < 0.001, partial η^2^ = 0.997, suggesting that LPC latency varied between the two recording devices. Furthermore, the Condition × Device interaction was significant, *F*(1, 24) = 6,027.00, *p* < 0.001, partial η^2^ = 0.996, indicating that the effect of condition on LPC latency differed across devices. *Post hoc* pairwise *t*-tests were conducted to explore the significant interaction between Condition and Device. The results showed that within the Biosemi system, there was no significant difference in LPC latency between the correct and incorrect conditions [*t*(24) = 1.91, *p* = 0.068, *d* = 0.38]. However, within the Neuphony system, LPC latency was significantly longer for correct trials compared to incorrect trials [*t*(24) = 2.71, *p* = 0.012]. When comparing devices, Neuphony showed significantly longer LPC latencies than Biosemi under both the correct [*t*(24) = −4.83, *p* < 0.001] and incorrect [*t*(24) = −3.91, *p* < 0.001] conditions. These findings suggest that device-related differences substantially influenced the latency of the LPC component, with consistently delayed responses observed in the Neuphony recordings. These results suggest that both task condition and recording device influenced the latency of the late positive complex, and that the pattern of latency differences between conditions was modulated by the device type.

## 4 Discussion

This study systematically evaluated the performance of the Neuphony EEG Flex Cap, a dry electrode system, against the BioSemi ActiveTwo, a laboratory-grade wet electrode system, under an experimentally controlled environment. Both systems were tested using identical 10–20 electrode montages during resting-state and task-based recordings to assess spectral characteristics and event-related potentials (ERPs) associated with key cognitive processes. Consumer EEG systems are frequently used for relaxation training, meditation, and pain or anxiety management (Ratti et al., 2017). Our results demonstrated that both systems successfully captured canonical EEG frequency bands, with comparable topographical distributions across conditions. The spectral power in alpha (8–13 Hz) and beta (13–30 Hz) bands commonly linked to relaxed wakefulness and cognitive engagement showed similar patterns between devices, confirming the dry system’s ability to record physiologically valid oscillatory activity. However, the Neuphony system exhibited slightly higher power in the lower frequency range (delta and theta). Particularly in wearable or portable EEG devices with dry electrodes and little shielding, lower frequency bands like delta and theta are more vulnerable to contamination from motion artifacts, baseline drift, and physiological noise (e.g., eye movements and perspiration) (Casson, 2019; Kappenman and Luck, 2010). In some bands, this may result in decreased fidelity and signal quality. On the other hand, systems designed for high-frequency detection, particularly those that use sophisticated filtering or denoising techniques, may better preserve or even enhance higher frequency components like beta and gamma, which are typically less affected by such artifacts (Lopez-Gordo et al., 2014; Krigolson et al., 2017). These differences, while statistically minor, indicate a modest reduction in signal-to-noise ratio at lower frequencies, likely due to mechanical instability at the skin–electrode interface and higher impedance variability inherent to dry sensors.

“Cognitive functions” is an umbrella term that includes a wide range of mental activities, such as perception, memory, and attention (Squires et al., 1977). These skills are typically thought to be strengthened by feedforward and feedback mechanisms and controlled by both top-down and bottom-up processes (Friedenberg et al., 2021). Hence, it is important to conduct EEG studies on various cognitive functions, including sustained attention, working memory, perceptual discrimination, etc., while evaluating consumer-grade EEG (Ratti et al., 2017). In this study, we covered a wide range of cognitive function-associated ERPs, especially those linked to attention and executive control, including both early and late potentials, along with oscillations during resting state, in order to get a multi-dimensional idea of the functioning of the newly developed device, which has not been previously done. In the visual discrimination and oddball paradigms, both devices successfully captured P300, MMN, and LPC components with comparable scalp topographies and polarity distributions. Despite comparable waveform profiles, systematic differences in amplitude were observed between devices. The Biosemi system produced slightly higher overall amplitudes, particularly for the oddball condition, likely reflecting the inherently lower impedance and optimized conductivity of wet electrodes. In contrast, the Neuphony system, while showing slightly attenuated amplitudes, maintained a consistent and well-defined P300 morphology across participants. This indicates that the dry-electrode configuration is capable of faithfully capturing the underlying neural dynamics despite potential variations in skin electrode contact. However, the latency of the P300 peak was delayed for Neuphony which is consistent with Bluetooth transmission lag and lower sampling synchronization precision compared to BioSemi’s fiber-optic acquisition (Rondon et al., 2019). Similarly, MMN and LPC components exhibited minor latency delays and slight amplitude attenuation, both within the range reported in previous dry vs. wet electrode comparisons (Krigolson et al., 2017; Ratti et al., 2017).

The Neuphony EEG Flex Cap demonstrated a more convenient setup, which aligns with its intended application in self-help contexts. Although it exhibited a higher proportion of artifact-contaminated epochs particularly at frontal sites, as reflected by lower SNR values at Fp1 and Fp2 (Table 1) which is likely attributable to increased sensitivity to facial muscle activity and minor electrode movement. While we did not objectively measure setup time or administer a formal comfort questionnaire, several participants informally reported that the dry headcap felt more comfortable and subjectively quicker to set up. These anecdotal reports suggest potential usability advantages, though they should be interpreted cautiously in the absence of systematic assessment. These findings align with reports emphasizing the trade-off between signal purity and convenience in portable EEG systems (Maskeliunas et al., 2016; Krigolson et al., 2017; Ratti et al., 2017). One other major factor contributing to the delay in latencies for ERPs obtained can be the integration of Bluetooth. While state-of-the-art devices like the BioSemi device utilize optical fibers for sending the EEG signals to the acquisition device, consumer-grade devices like the Neuphony flex cap rely on Bluetooth for sending the signals, which is again not a very fair comparison. Positively, Bluetooth-based EEG devices have a lot to offer in terms of scalability and portability. These systems allow for wireless data transmission, which removes the need for large hardware configurations and makes way for more efficient, app-based EEG acquisition platforms (Casson, 2019; Looney et al., 2012). They are especially well-suited for use in distant or field-based environments, such as defense applications, high-altitude physiology research, or home-based neurocognitive monitoring, because of their increased mobility (Stopczynski et al., 2014; Krigolson et al., 2017). The scope and viability of longitudinal and ecologically sound neuroscience research are significantly increased by the capacity to conduct EEG recordings outside of conventional lab or hospital settings.

According to a number of studies, signals captured with dry electrodes are noisier (Johnstone et al., 2012; Halford et al., 2016; Clements et al., 2016; Cruz-Garza et al., 2017; Spüler, 2017). Our findings that the dry EEG device produced more artifact segments and delays in ERP for tasks are in line with this. Additionally, the dry EEG system’s higher power in low frequencies (Delta and partly Theta) (Figure 2) suggests underlying artifact-related signal oscillations. With very minor variations in lower frequency bands, the similar spatial distribution of all band power levels further supports the idea that the EEGs obtained from the two devices while the eyes are open and at rest are comparable. Similarly, ERPs obtained like P300, MMN and LPCs followed the same trend across both devices, with minor delay and decreased sensitivity for Neuphony EEG flex cap that might be attributed to the use of dry sensors (Johnstone et al., 2012; Halford et al., 2016) and the use of Bluetooth (Rondon et al., 2019). Due to intrinsic restrictions in bandwidth, data transfer, and power efficiency, Bluetooth-based EEG systems restrict signal sensitivity even while they allow wireless and portable recordings (Casson, 2019; Lopez-Gordo et al., 2014). Lower sample rates, fewer channels, or signal compression are frequently required due to the limited bandwidth, which may jeopardize the precise capture of high-frequency components and delicate brain dynamics (Krigolson et al., 2017). Furthermore, power-saving measures like onboard filtering and lower-resolution analog-to-digital conversion may weaken low-frequency signals or decrease amplitude precision (Kappenman and Luck, 2010). The temporal fidelity of event-related potential is further impacted by transmission instabilities such as timing jitter or packet loss (Casson, 2019). When evaluating the validity of Bluetooth-enabled EEG devices against clinical-grade systems, it is important to take into account the fact that these factors can collectively decrease their sensitivity, especially when it comes to detecting high-frequency or fine-grained neural activity. The reliability and validity of the Bluetooth for EEG signal acquisition may provide additional insights into the usability of the EEG device in different settings.

In an experimentally controlled setting, we compared dry and wet EEG systems based on the 10–20 electrode montage. Because no direct spectral quantification of 50 Hz interference was performed in the present study, we cannot draw empirical conclusions regarding differences in power-line noise between the two devices. Although Neuphony incorporates active shielding circuitry designed to reduce capacitive coupling and environmental electromagnetic interference, and BioSemi’s CMS/DRL system is optimized for common-mode noise suppression, the present dataset does not allow us to evaluate whether these hardware differences result in measurable variations in line-noise susceptibility. Prior work has suggested that certain dry-electrode systems can exhibit reduced sensitivity to ambient electromagnetic interference (Pourahmad and Mahnam, 2016), suggesting that it is less susceptible to electromagnetic interference from background noise that may be present at home or at a clinic. When combined, these findings imply that the dry EEG technology, shows promise and merits more research to determine its suitability for use in domestic settings.

Taken together, these findings indicate that Neuphony’s dry electrode technology can reliably reproduce core EEG and ERP signatures obtained with a research-grade system, despite expected limitations in latency precision and low-frequency noise susceptibility. The comparable P300, MMN, and LPC amplitudes, along with consistent alpha–beta power distributions, demonstrate that the dry system can capture meaningful neural responses across both sensory and cognitive domains. The portability, ease of setup, and participant preference highlight its promise for large-scale, home-based, or ecologically valid recordings, particularly where rapid deployment and user comfort are critical. While laboratory-grade systems like BioSemi remain the gold standard for high-density, high-fidelity EEG acquisition, wearable systems such as Neuphony expand the methodological frontier by enabling continuous, real-world monitoring of cognitive states. Due to the heavy and stationary equipment, traditional laboratory investigations may have lower ecological validity and participant discomfort, even while offering highly controlled settings and greater signal quality. Continuous monitoring during daily tasks is made possible by wearable EEG devices, which give researchers the opportunity to investigate the neural correlates of workload, attention, and cognition in realistic environments. Important neurological fingerprints in ambulatory participants were highlighted by seminal studies by Debener et al. (2012), Mullen et al. (2015), and Makeig et al. (2010), which showed that mobile EEG is feasible for recording event-related potentials and oscillatory activity outside of the lab. In a similar vein, Mullen et al. (2015) demonstrated that wireless, lightweight EEG equipment may effectively monitor cognitive load and fatigue in practical settings. With its reasonable signal integrity, mobility, and user comfort, the Neuphony wearable EEG offers a balance to the high-density, stationary setup of the BioSemi system. Consistent with this direction, our findings support the feasibility of the Neuphony EEG Flex Cap for research and applied neurophysiology.

Although the present study involves comparison between a dry-electrode system and a laboratory-grade wet-electrode system, its contribution extends beyond device validation. With the rapid expansion of EEG use in real-world and semi-naturalistic environments, there is a critical need to determine whether emerging dry-electrode systems can capture cognitively relevant neural signatures with the same fidelity as established research-grade equipment. The P300, MMN, and LPC components are among the robust electrophysiological markers of attention, stimulus discrimination, and cognitive evaluation. Demonstrating whether these components can be extracted reliably using a wearable dry-electrode system is therefore essential not only for device development but also for expanding the methodological toolbox available to cognitive neuroscience, psychophysiology, and applied neuro ergonomics. By formally quantifying amplitude, latency, signal-to-noise ratio, and spectral power differences across systems, this study provides evidence about the feasibility and limitations of dry-electrode EEG for future experimental and translational research. Our comparison of BioSemi and Neuphony demonstrates that although the conventional system is superior in terms of signal quality and spatial resolution, wearable technology allows for flexible, longitudinal, and field-based research, greatly expanding the range and usefulness of neurophysiological studies outside of the lab. Nonetheless, future work should further examine signal stability across longer sessions, ambient noise resilience, and cross-environment reliability to fully establish its validity for clinical and field applications.

## Data Availability

The original contributions presented in the study are included in the article/supplementary material, further inquiries can be directed to the corresponding author/s.
